# Longitudinal levels and bouts of sedentary time among adolescent girls

**DOI:** 10.1186/1471-2431-13-173

**Published:** 2013-10-21

**Authors:** Valerie Carson, Dylan P Cliff, Xanne Janssen, Anthony D Okely

**Affiliations:** 1 Faculty of Physical Education and Recreation, University of Alberta, Edmonton, AB, Canada; 2 Interdisciplinary Educational Research Institute, Faculty of Education, University of Wollongong, Wollongong, NSW, Australia

**Keywords:** Sedentary time, Patterns, Adolescents, Girls

## Abstract

**Background:**

Adolescent girls are one of the most sedentary demographic groups. A better understanding of their accumulation of sedentary time is needed to inform future interventions. The purpose of this study was to examine the longitudinal levels and bouts of objectively measured sedentary time accumulated during different days of the week and periods of the weekday among a large sample of adolescent girls.

**Methods:**

The results are based on 655 adolescent girls from the Girls in Sport Intervention and Research Project. Levels and bouts of sedentary time were derived from accelerometer data collected at baseline and 18-month follow-up. Total, weekday, weekend, school (i.e., morning bell to afternoon bell), after school (i.e., afternoon bell to 19:00), and evening (i.e. 19:01 to 23:59) sedentary time levels and bouts were calculated. Repeated-measures ANCOVAs were conducted to examine differences in sedentary time levels and bouts between days and time periods after adjusting for wear time, accelerometer model, and intervention group.

**Results:**

Cross-sectional analyses revealed that levels and bouts of sedentary time were higher on weekdays compared to weekend days at baseline. Similar trends were observed at follow-up. In addition, percentage of wear time spent sedentary and bouts/hr of sedentary time were highest in the evening compared to the school and after school periods at both baseline and follow-up. Longitudinal analyses revealed that levels and bouts of sedentary time were higher at follow-up compared to baseline across the different days of the week and periods of the weekday examined, with the biggest increase (15%) occurring in the school period.

**Conclusions:**

Future interventions targeting sedentary time among adolescent girls should consider developing strategies to reduce and break up prolonged sedentary time during the school day and in the evening.

## Background

Sedentary behavior is increasingly being recognized as an important area of study in health research. Sedentary behavior can be defined as any waking behavior characterized by an energy expenditure ≤1.5 METs while in a sitting or reclining posture [1]. Sedentary behavior is considered distinct from a lack of moderate- to vigorous-intensity physical activity (MVPA) [1,2]. For instance, excessive screen-based sedentary behavior is linked with obesity and related cardio-metabolic risk factors independent of MVPA [3,4]. While findings of existing studies between objectively-measured sedentary behavior and young people’s health have been inconsistent [3,5-8], sedentary behaviors among young people may track into adulthood [9]. Among adults, independent associations have been observed between objectively measured levels of sedentary time - as well as patterns of sedentary time - (i.e., prolonged bouts versus shorter broken up bouts) and cardio-metabolic health [10-14]. Therefore, targeting levels and patterns of sedentary time among young people may have important short- and long-term health benefits.

Data from several countries indicate that adolescents spend a large portion of waking hours sedentary [15-17]. In fact, national data from the United States show that adolescents (ages 12–18 years) are one of the most sedentary age groups [16]. Furthermore, among adolescents, girls are more sedentary than boys [15-17]. Therefore, adolescent girls are a potentially important target group for future sedentary behavior interventions. Further evidence regarding the accumulation of sedentary time for different days of the week, and different periods of the weekday, among adolescent girls could help inform future targeted interventions. To date, little work has examined the accumulation of objectively-measured sedentary time among adolescent girls. One cross-sectional study found that levels of sedentary time were higher by 142 min/day or 5% of wear time on weekdays compared to weekend days among 1603 adolescent girls [18]. Conversely, another cross-sectional study among 111 adolescent girls [19] did not find differences in levels of sedentary time between weekday and weekend days or between during school and after school time periods. In terms of patterns of sedentary time, this same study found that longer bouts of sedentary time were accumulated on weekdays compared to weekend days and during school compared to after school [19]. However, the results of this study may not be generalizable due to the small sample size.

Another limitation of the available evidence examining the accumulation of objectively-measured sedentary time among adolescent girls is that the aforementioned studies both reported on cross-sectional data. If levels and bouts of sedentary time are found to increase during adolescence, this would provide further support for the development of targeted intervention approaches. To our knowledge only one study has examined the longitudinal levels of adolescent girls’ objectively-measured sedentary time. Treuth and colleagues found levels of sedentary time among girls increased by 51 mins/day from 6th (461 min/day) to 8th grade (512 min/day) [20]; however, it is unknown whether these increases tend to be consistent across different days of the week or periods of the weekday. Furthermore, it is unknown if patterns of sedentary time such as bouts, change during adolescence. Therefore, the purpose of this study was to examine the longitudinal levels and bouts of objectively measured sedentary time accumulated during different days of the week and different periods of the weekday among a large sample of adolescent girls.

## Methods

### Participants

Data for this study were collected as part of the Girls in Sport Intervention and Research Project. This was a school-based 18-month randomized control trial aimed at preventing the decline of accelerometer-derived MVPA among adolescent girls in New South Wales, Australia. Baseline data were collected among 12- to 15-year olds in 24 schools from February to June 2009. Following data collection, 12 intervention schools participated in the Girls in Sport program, which involved school-specific action plans targeting school sport, promoting physical activity during break times, and linking with sport and physical activity organizations in the local community. The 12 control schools continued with their regular school programs. Follow-up data were collected from July to December 2010. More detail about the study, including its methods, can be found elsewhere [21]. Ethics approval was obtained from the University of Wollongong Human Research Ethics Committee. Informed consent was also obtained from the NSW Department of Education and Communities, participating schools, parents, and students.

A total of 86% of eligible girls completed baseline assessments resulting in 1518 participants. Since the intervention targeted MVPA and had no effect on sedentary behavior (adjusted difference between groups [Int-Ctl] = −2.81 mins/day; 95% CI [−21.74, 16.19]; P = 0.87) the intervention and control groups were combined for all analyses. Complete accelerometer data (as explained below) were available for 1262 participants at baseline. A total of 1241 girls completed follow-up assessments and complete accelerometer data was available for 721 participants. In total, 655 participants had complete accelerometer data for both baseline and follow-up and were included in the final sample. There were no significant differences in baseline age, follow-up age, and baseline levels of sedentary time between those participants included and excluded in the final sample (P > 0.05). However, levels of sedentary time at follow-up were 2.5% higher for those participants excluded from the final sample compared to those included (P < 0.05). Analyses involving weekend days were conducted in the 570 participants at baseline, 415 at follow-up, and 381 at both baseline and follow-up that had ≥1 valid weekend days (as explained below). After adjusting for wear time, no significant differences in levels of sedentary time were observed at both time points between participants that had and did not have ≥1 valid weekend day (P > 0.05).

### Demographics

Participants’ age was recorded at both baseline and follow-up. Ethnicity was assessed by asking participants what country their mother was born in. Parental education was also assessed at follow-up by asking participants what their mother’s highest level of schooling was. There were 7 response options ranging from ‘no formal education’ to ‘postgraduate qualifications’.

### Sedentary time

Levels and bouts of sedentary time were derived from accelerometer data. Participants wore Actigraph accelerometers (7164 and GT1M models; For Walton Beach, FL) on their right hip during waking hours for seven consecutive days at baseline and follow-up using an adjustable elastic belt. Participants were given the same accelerometer model at both time-points. Average intensities over 30-second epochs were recorded. Non-wear time was defined as a period of >60 minutes of zero counts [22]. Only participants with a minimum of 10 hours of wear time per day for three days were included in the analyses. A cut point of ≤ 100 counts per minute (≤ 50 counts per minute/30 seconds) was used to define sedentary time. This cut point, which is commonly used in the literature [16], has been validated in adolescent girls [23], and has been shown to exhibit the highest classification accuracy among youth [24]. Total, weekday, weekend, school (i.e., morning bell to afternoon bell), after school (i.e., afternoon bell to 19:00), and evening (i.e. 19:01 to 23:59) sedentary time was calculated. Sedentary time was expressed as minutes per day and as a percentage of wear time to account for the different total minutes in the school, after school, and evening periods. Wear time was specific to the period being examined. Furthermore, total, weekday, weekend, school, after school, and evening sedentary bouts lasting 10, 20, and 30 minutes were calculated. A bout was defined as a continuous period of sedentary time and the bout stopped when the counts for a 30-sec epoch went above the sedentary time cut point. Sedentary bouts were expressed as bouts per day and bouts per hour to account for the different total minutes in the school, after school, and evening periods.

### Data analysis

Analyses were completed using SAS version 9.2 (SAS Institute Inc., Cary, NC). Descriptive statistics were calculated including means and standard deviations for levels and bouts of sedentary time at both baseline and follow-up. The MIXED procedure was used to calculate repeated-measures ANCOVAs to examine cross-sectional differences in levels and bouts of sedentary time between weekdays and weekend days and between school, after school, and evening periods at both measurement periods. Additionally, repeated-measures ANCOVAs were conducted to examine changes in levels and bouts of sedentary time over the two measurement periods. All ANCOVAs analyses were adjusted for wear time, accelerometer model, and intervention group (i.e., control versus intervention). Finally, the tracking of total, weekday, and weekend levels of sedentary time were examined by ranking participants with valid baseline and follow-up data and calculating Spearman rank-order correlation coefficients.

## Results

The average age of participants was 13.5 (0.4 SD) years at baseline and 14.9 (0.4 SD) years at follow-up. The majority of the sample (91.8%) had a mother born in Australia. In terms of mothers’ education, 4.0% had no formal education, 20.2% had Grade 10, 15.9% had Grade 12, 7.9% had trade/apprentice, 6.3% had a diploma, 14.4% had University, 6.1% had postgraduate, and 25.2% were missing/don’t know. Valid days of wear and valid wear time were similar across assessment time points, with the exception of valid wear time during school, which was approximately 90 min/day higher at baseline and valid wear time after school, which was approximately 40 min/day higher at follow-up.

Total, weekday, and weekend levels of sedentary time are presented in Table 1. The average levels of sedentary time were 518.9 min/day (63.1% of wear time) at baseline and 545.3 min/day (67.6% of wear time) at follow-up. Participants engaged in significantly more sedentary time on weekdays compared to weekend days at baseline (P < 0.05). The same trend was seen at follow-up; however, the differences did not reach statistical significance. When comparing baseline to follow-up assessments, total, weekday, and weekend levels of sedentary time were all significantly higher at follow-up. For tracking of sedentary time, moderate correlations were observed for total (r = 0.45 for min/day and r = 0.55 for percentage of wear time), weekday (r = 0.45 for min/day and r = 0.54 for percentage of wear time), and weekend (r = 0.32 for min/day and r = 0.41 for percentage of wear time) levels of sedentary time.

**Table 1:** Total, weekday, and weekend objectively measured sedentary time

	**Total**		**Weekdays**		**Weekends**	
	**(N = 655)**		**(N = 655)**		**(N = 381)**	
	**Baseline**	**Follow-up**	**Baseline**	**Follow-up**	**Baseline**	**Follow-up**
Min/day	518.9 (92.0)	545.3 (82.3)^b^	526.8 (90.1)	551.3 (82.2)^b^	496.0 (137.2)	529.6 (137.7)^a,b^
Percentage of wear time	63.1 (0.1)	67.6 (0.1)^b^	63.2 (0.1)	67.8 (0.1)^b^	62.5 (0.1)	67.2 (0.1)^a,b^

Data are presented as mean (standard deviation) and p-values were adjusted for wear time, type of device, and intervention group.

^a^Significant cross-sectional differences between weekday and weekend at baseline (N = 570; P < 0.05).

^b^Significant longitudinal differences between baseline and follow-up (P < 0.05).

School, after school, and evening levels of sedentary time are presented in Table 2. When examining percentage of wear time, participants engaged in the most sedentary time during the evening period at both baseline (69.0%) and follow-up (74.2%). Participants at baseline engaged in significantly more sedentary time after school compared to during school, (58.6 versus 54.1%); whereas, participants at follow-up engaged in significantly more sedentary time during school compared to after school (69.3 versus 63.9%). When comparing baseline to follow-up, levels of sedentary time were significantly higher at follow-up for all three periods of the day. This was most prominent for the school period where participants’ percentage of wear time spent sedentary increased from 54.1% at baseline to 69.3% at follow-up.

**Table 2:** During school, after school, and evening objectively measured sedentary time

	**School**		**After school**		**Evening**	
	**(N = 655)**		**(N = 655)**		**(N = 655)**	
	**Baseline**	**Follow-up**	**Baseline**	**Follow-up**	**Baseline**	**Follow-up**
Min/day	243.1 (35.7)	256.7 (34.4)^c^	132.0 (27.8)	137.2 (30.0)^c^	252.6 (51.8)	281.7 (89.3)^c^
Percentage of wear time	54.1 (0.1)	69.3 (0.1)^c^	58.6 (0.1)	63.9 (0.1)^c^	69.0 (0.1)	74.2 (0.1)^a,b,c^

Data are presented as mean (standard deviation) and p-values were adjusted for wear time, type of device, and intervention group.

School = Morning bell to afternoon bell; After school = Afternoon bell to 19:00; Evening = 19:01 to 23:59.

^a^Significant cross-sectional difference between time periods for percentage of wear time at baseline (P < 0.05).

^b^Significant difference between time periods for percentage of wear time at follow-up (P < 0.05).

^c^Significant longitudinal difference between baseline and follow-up (P < 0.05).

Total, weekday, and weekend sedentary bouts are presented in Table 3. As the length of the bout increased, the number of bouts/hr and bouts/day decreased at both baseline and follow-up. There was a significantly higher number of bouts/hr and bouts/day on weekdays compared to weekend days for all bout lengths at baseline. The same trend was seen at follow-up; however, it did not reach statistical significance. When comparing baseline to follow-up assessments, there were significantly more total, weekday, and weekend bouts/hr and bouts/day at follow-up for all bout lengths. For example, at baseline girls averaged approximately one 30 min bout/10 hr, while at follow-up this had increased to approximately two 30 min bouts/10 hr.

**Table 3:** Number of total weekday and weekend sedentary bouts

		**Total**		**Weekdays**		**Weekends**	
		**(N = 655)**		**(N = 655)**		**(N = 381)**	
		**Baseline**	**Follow-up**	**Baseline**	**Follow-up**	**Baseline**	**Follow-up**
10 mins	Bouts/hr	1.22 (0.46)	1.60 (0.53)^b^	1.23 (0.53)	1.61 (0.53)^b^	1.14 (0.63)	1.51 (0.77)^a,b^
	Bouts/day	16.83 (7.15)	21.52 (7.87)^b^	17.27 (7.23)	21.84 (7.87)^b^	15.37 (10.25)	20.28 (12.12)^a,b^
20 mins	Bouts/hr	0.30 (0.16)	0.44 (0.21)^b^	0.31 (0.17)	0.45 (0.21)^b^	0.27 (0.23)	0.40 (0.34)^a,b^
	Bouts/day	4.19 (2.45)	5.94 (3.00)^b^	4.31 (2.48)	6.05 (2.98)^b^	3.74 (3.77)	5.48 (4.87)^a,b^
30 mins	Bouts/hr	0.12 (0.08)	0.18 (0.11)^b^	0.12 (0.08)	0.18 (0.11)^b^	0.10 (0.12)	0.16 (0.18)^a,b^
	Bouts/day	1.60 (1.19)	2.43 (1.56)^b^	1.64 (1.20)	2.47 (1.56)^b^	1.45 (1.95)	2.24 (2.59)^a,b^

Data are presented as means (standard deviation) and p-values are adjusted for wear time, type of device, and intervention group

^a^Significant cross-sectional differences between weekday and weekend at baseline (N = 570; P < 0.05).

^b^Significant longitudinal differences between baseline and follow-up (P < 0.05).

School, after school, and evening sedentary bouts are presented in Table 4. As the length of bout increased the number of bouts/hr and bouts/day decreased at both baseline and follow-up. When examining bouts/hr, participants engaged in the most sedentary bouts in the evening, followed by the school period, at both baseline and follow-up. When comparing baseline to follow-up assessments, bouts/hr and bouts/day were significantly higher at follow-up for all three periods of the day (Table 4).

**Table 4:** Number of during school, after school, and evening sedentary bouts

		**School**		**After school**		**Evening**	
		**(N = 655)**		**(N = 655)**		**(N = 655)**	
		**Baseline**	**Follow-up**	**Baseline**	**Follow-up**	**Baseline**	**Follow-up**
10 mins	Bouts/hr	1.12 (0.52)	1.70 (0.68)^c^	1.01 (0.57)	1.37 (0.69)^c^	1.52 (0.77)	1.99 (0.90)^a,b,c^
	Bouts/day	8.35 (3.69)	10.49 (4.23)^c^	3.78 (2.13)	4.88 (2.46)^c^	3.85 (2.41)	4.95 (2.87)^c^
20 mins	Bouts/hr	0.29 (0.19)	0.48 (0.28)^c^	0.23 (0.21)	0.36 (0.30)^c^	0.38 (0.32)	0.58 (0.41)^a,b,c^
	Bouts/day	2.15 (1.38)	2.96 (1.74)^c^	0.84 (0.76)	1.24 (0.93)^c^	0.97 (0.88)	1.42 (1.08)^c^
30 mins	Bouts/hr	0.11 (0.10)	0.20 (0.15)^c^	0.08 (0.10)	0.14 (0.15)^c^	0.15 (0.17)	0.23 (0.23)^a,b,c^
	Bouts/day	0.82 (0.70)	1.22 (0.94)^c^	0.29 (0.36)	0.49 (0.49)^c^	0.38 (0.46)	0.58 (0.58)^c^

Data are presented as mean (standard deviation) and p-values are adjusted for wear time, type of device, and intervention group.

School = Morning bell to afternoon bell; After school = Afternoon bell to 19:00; Evening = 19:01 to 23:59.

^a^Significant cross-sectional difference between time periods for percentage of wear time at baseline (P < 0.05).

^b^Significant difference between time periods for percentage of wear time at follow-up (P < 0.05).

^c^Significant longitudinal difference between baseline and follow-up (P < 0.05).

## Discussion

This study examined the longitudinal levels and bouts of objectively measured sedentary time accumulated during different days of the week and different periods of the weekday among adolescent girls. Cross-sectional analyses revealed that levels and bouts of sedentary time were higher on weekdays compared to weekend days at baseline. Similar trends were observed at follow-up. In addition, percentage of wear time spent sedentary and bouts/hr of sedentary time were highest in the evening compared to the school and after school periods at both baseline and follow-up. Longitudinal analyses revealed that levels and bouts of sedentary time were higher at follow-up compared to baseline across the different days of the week and periods of the weekday examined, with the biggest increase (15%) occurring in the school period. Furthermore, total, weekday, and weekend levels of sedentary time tracked moderately across the two measurement periods.

The finding that this sample of Australian adolescent girls spent a large proportion of their waking hours sedentary at both baseline (518.9 min/day or 63.1% of wear time) and follow-up (545.3 min/day or 67.6% of wear time) is consistent with previous work showing adolescent girls are a particularly sedentary demographic group [15-17]. The baseline findings, when girls were aged 13.5 years, are slightly lower than previous cross-sectional estimates among 11–14 year-old girls in Canada (527 min/day or 65% of wear time) [17], and 12.5-13.5 year-old girls from several European countries (534 min/day or 70% of wear time) [15] but higher compared to 12–15 year-old girls in the United States (462 min/day or 55% of wear time) [16]. These differences may be explained by different data reduction techniques, such as definitions of non-wear time, which were used in the studies. One novel aspect of the present study is the examination of longitudinal levels of objectively measured sedentary time and increases were observed over an 18-month period. Only one other study to our knowledge has done a similar analysis in this demographic group. Consistent with the present study, Treuth and colleagues reported a 51 min/day increase in total levels of sedentary time among approximately 984 12-to 14-year old girls over a 2-year period [20]. While this increase in sedentary time was larger than the present study, the follow-up period was also longer. Combined these findings suggest that adolescence is characterized by an increase in sedentary time among girls. We also observed that sedentary levels tracked moderately over the 18-month period; therefore, early adolescence among girls may be a critical period for targeting sedentary time.

Another novel aspect of the present study is the examination of both cross-sectional and longitudinal levels and bouts of sedentary time during different days of week and periods of the weekday to provide information on day or time periods to target for future interventions. In the present study, levels and bouts of sedentary time were slightly higher on weekdays compared to weekend days at baseline, and similar trends were observed at follow-up. While statistical significance was reached at baseline, these findings might not be of practical significance. However, previous studies using objective measures of sedentary time among adolescents have observed higher levels of sedentary behavior (62 min/day) [25], or more sedentary bouts (4/day) [19] on weekdays than weekend days that were practically significant.

When examining changes in levels of sedentary time across different day and time periods, the largest increase (~15%) was observed for the during school time period compared to an increase of approximately 5% for weekend, weekday, after school, and evening measurements. These findings suggest that the school environment might be influential in shaping girls’ levels and patterns of sedentary time. Therefore, future interventions that aim at reducing and breaking up prolonged sitting in the school setting should be considered. These interventions could incorporate strategies such as standing lessons and short activity breaks [26].

In addition to the school environment, the home environment, particularly in the evening, may also influence girls’ sedentary time, given the finding that the percentage of wear time spent sedentary and bouts/hr were highest in the evening time period at both baseline and follow-up. Therefore, in addition to the school setting, future interventions should also consider developing strategies to break up sedentary time during the evening in the home environment. Future research is needed to evaluate interventions targeting school and evening sedentary time among adolescent girls to determine the most appropriate strategies for sedentary time reduction.

Strengths of this study include the objective measure of sedentary time as well as the unique analysis of changes in levels and patterns during different segments of the day, which identified specific time periods that could potentially be targeted for future interventions. A limitation of the study includes the use of two different types of accelerometer models. However, some evidence suggests that differences between models may be small [27,28]. Likewise, participants received the same accelerometer model at baseline and follow-up, and all analyses adjusted for monitor types.

## Conclusion

Levels and bouts of sedentary time significantly increased over an 18-month period in a large sample of adolescent girls. The largest increase in levels of sedentary time was observed during school. Furthermore, the evening time period had the highest levels of sedentary time and the most sedentary bouts at both baseline and follow-up. Thus, future interventions targeting levels and patterns of sedentary time among adolescent girls should consider developing strategies to reduce and break up sedentary time during school and in the evening.

## Competing interests

The authors declare that they have no competing interests.

## Authors’ contributions

ADO took the lead in designing the study and secured the funding. VC performed the statistical analyses. All authors interpreted the data. VC wrote the manuscript. All authors critically reviewed and revised the manuscript for important intellectual content. All authors read and approved the final version of the manuscript.

## Pre-publication history

The pre-publication history for this paper can be accessed here:

http://www.biomedcentral.com/1471-2431/13/173/prepub
